# Natural Language Processing of Referral Letters for Machine Learning–Based Triaging of Patients With Low Back Pain to the Most Appropriate Intervention: Retrospective Study

**DOI:** 10.2196/46857

**Published:** 2024-01-30

**Authors:** Sebastian Fudickar, Carsten Bantel, Jannik Spieker, Heinrich Töpfer, Patrick Stegeman, Henrica R Schiphorst Preuper, Michiel F Reneman, André P Wolff, Remko Soer

**Affiliations:** 1 Institute of Medical Informatics University of Lübeck Lübeck Germany; 2 Department of Medicine and Public Health University of Oldenburg Oldenburg Germany; 3 University Clinic for Anesthesiology, Intensive Care Medicine, Emergency Medicine and Pain Therapy University of Oldenburg Oldenburg Germany; 4 University Oldenburg Oldenburg Germany; 5 Department of Rehabilitation Medicine University Medical Center Groningen University of Groningen Groningen Netherlands; 6 Department of Anesthesiology University Medical Center Groningen University of Groningen Groningen Netherlands; 7 Research Group Smart Health Saxion University of Applied Sciences Enschede Netherlands

**Keywords:** decision support, triaging, NLP, natural language processing, neural network, LBP, low back pain, back, pain, decision-making, machine learning, artificial intelligence, clinical application, patient records, qualitative data, support system, questionnaire, quality of life, psychosocial

## Abstract

**Background:**

Decision support systems (DSSs) for suggesting optimal treatments for individual patients with low back pain (LBP) are currently insufficiently accurate for clinical application. Most of the input provided to train these systems is based on patient-reported outcome measures. However, with the appearance of electronic health records (EHRs), additional qualitative data on reasons for referrals and patients’ goals become available for DSSs. Currently, no decision support tools cover a wide range of biopsychosocial factors, including referral letter information to help clinicians triage patients to the optimal LBP treatment.

**Objective:**

The objective of this study was to investigate the added value of including qualitative data from EHRs and referral letters to the accuracy of a quantitative DSS for patients with LBP.

**Methods:**

A retrospective study was conducted in a clinical cohort of Dutch patients with LBP. Patients filled out a baseline questionnaire about demographics, pain, disability, work status, quality of life, medication, psychosocial functioning, comorbidity, history, and duration of pain. Referral reasons and patient requests for help (patient goals) were extracted via natural language processing (NLP) and enriched in the data set. For decision support, these data were considered independent factors for triage to neurosurgery, anesthesiology, rehabilitation, or minimal intervention. Support vector machine, k-nearest neighbor, and multilayer perceptron models were trained for 2 conditions: with and without consideration of the referral letter content. The models’ accuracies were evaluated via F1-scores, and confusion matrices were used to predict the treatment path (out of 4 paths) with and without additional referral parameters.

**Results:**

Data from 1608 patients were evaluated. The evaluation indicated that 2 referral reasons from the referral letters (for anesthesiology and rehabilitation intervention) increased the F1-score accuracy by up to 19.5% for triaging. The confusion matrices confirmed the results.

**Conclusions:**

This study indicates that data enriching by adding NLP-based extraction of the content of referral letters increases the model accuracy of DSSs in suggesting optimal treatments for individual patients with LBP. Overall model accuracies were considered low and insufficient for clinical application.

## Introduction

Triaging patients with low back pain (LBP) for the best-fitting treatment is a complex interaction between evidence-based standard application, clinical reasoning, and patient preferences. Many factors interact within the biopsychosocial model for LBP, making it difficult to provide the best-possible treatment by the right professional based on the best evidence. In general, the pain of 95% of patients with LBP is considered nonspecific, meaning that a clear somatic cause for pain cannot be determined [1]. The diagnosis of LBP, therefore, is often insufficient to choose the best treatment modality. Within the broad spectrum of complaints and the lack of a clear-cut diagnosis, conservative treatments, such as interdisciplinary rehabilitation or other activating therapies, are considered to have the highest level of evidence [2]. Other treatment options, including anesthesiology or surgery, may be indicated for subgroups. However, the complex biopsychosocial interactions make identifying which patient may require which treatment difficult.

Attempts have been made to group patients with overlapping characteristics to individualize treatment [3]. Groups have been established based on psychosocial characteristics (among others, the Örebro Musculoskeletal Pain Questionnaires [4]), case complexity (eg, the Start Back Screening Tool [5]), or, for example, clinical prediction rules [6]. These subgrouping studies are a step forward in providing patients with targeted treatment based on their characteristics. Although the generalization of these models appears limited, they are not easily adaptable to other contexts. They are insufficiently capable of stratifying treatment based on a possibly infinite number of patient characteristics.

Health care professionals have been able to triage patients to surgical, anesthesiologic, or rehabilitation interventions with moderate agreement [7]. To progress in individualizing treatment and referrals, artificial intelligence systems, particularly machine learning (ML), are a relatively new method that can identify patient patterns based on more input than can be incorporated into traditional models. A few examples have appeared on LBP previously, showing that models could be applied to the data [7] and support the decision of who should get low back surgery [8]. A clinical decision support system (CDSS) may lead to an increase in triaging patients to the best-possible or targeted treatment and increase the cost-effectiveness of current care for LBP. These models, however, are limited to self-report questionnaires as input and may consequently still have low model fit values, leading to nonvaluable support tools [7].

Electronic health records (EHRs) potentially contain a huge source of qualitative data, including scans, referral information, and other free-text information data, which may increase the information quality as input for a CDSS. The data’s value, however, for the triaging process of patients with LBP and how these data can be enriched are unknown.

Therefore, this study aimed to investigate the added predictive value of qualitative referral letter information for ML-based triaging of patients with LBP to either neurosurgery, anesthesiology, rehabilitation, or a minimal intervention based on information and education.

## Methods

### Recruitment

A retrospective study was conducted in a clinical cohort of patients with chronic LBP. Patients referred to the Groningen Spine Center, a university-based tertiary center, were invited to participate. To be eligible for participation, patients had to be at 18 years or older and need to report predominantly LBP, either with or without radiation to the legs. We decided not to exclude patients based on comorbidity, life expectancy, work, or health status to reflect daily care. The Groningen Spine Center is a multidisciplinary center comprising, among others, rehabilitation, neurosurgery, and anesthesiology departments. Prior to participation, patients received informative material on the purpose of a larger 10-year prospective cohort study (the Groningen Spine Cohort Study [9]) to study long-term outcomes with LBP. This study was part of the cohort study.

### Ethical Considerations

Patients provided signed informed consent. The Medical Ethical Committee of the University Medical Center Groningen provided a waiver for this study because no other data other than care as usual were being used (M15.169472). The research followed the Declaration of Helsinki and the Good Clinical Practice.

### Clinical Procedures and Measures

After referral by a general practitioner or a second-line specialist, the referral letters were automatically stored in the spine center’s EHRs (Hyperspace version February 2022, Epic Systems). The retrieved referral letters were digitized as text. After enrollment, patients were sent an online set of questionnaires to screen for potential impact, pain intensity, disability, and psychosocial factors that have been described to be correlated with LBP. All questionnaires were validated on LBP. The quality of life was assessed using the EQ-5D [10] Pain Impact, and patient characteristics, medical history, and symptoms and functioning were gathered using the National Institutes of Health (NIH) minimal data set for LBP [11]. Work ability was measured using the Work Ability Score (WAS [12]), and the psychosocial work environment was measured using the short Copenhagen Psychosocial Questionnaire (COPSOQ II [13]). Further details of the study characteristics are described in the study by Dutmer et al [9]. If patients were unable to fill out the questionnaires online, paper versions were sent by mail.

Prior to the first consultation, all patients were triaged by 1 of 4 physician assistants (PAs) specifically educated in spinal disorders. For this study, PAs’ referrals to rehabilitation, anesthesiology, surgery, or others consisted of education and advice, no treatment, further diagnostics, or referral to primary care (see Table 1 for details of the corresponding categorization). These referrals were considered our primary outcome, whereas the baseline questionnaires and EHR data were our predicting features. In addition to these established questionnaires, we considered the textual content of the digitized referral letters. With this dependency on the availability of digitized referral letters, cases without referral letters were not considered and were excluded. In the EHR data, we searched for the referral reason as well as the patient’s question for help, which was reported as the patient goal in this study.

**Table 1:** Considered referral categories and corresponding data entries (N=1209).

Referral category	Training set^a^ samples (pre-SMOTE), n (%)	Evaluation set samples, n (%)
Rehabilitation	521 (43.1)	50 (4.1)
Anesthesiologic pain therapy	178 (14.7)	50 (4.1)
Neurosurgery	59 (4.9)	50 (4.1)
Other treatment^b^	251 (20.8)	50 (4.1)

^a^The training set was balanced afterward via the synthetic minority oversampling technique (SMOTE), resulting in each class holding 521 samples.

^b^“Other treatment” represents a minimal intervention based on information material.

#### Data Preprocessing

The following preprocessing steps were applied to the data. A data set including all questionnaires was enriched with additional referral letter parameters (see the following section). The selection of multiple referral reason or patient goal features/answers per letter was supported. However, if patients had multiple referral letters, only the referral reasons and patient goal of the initial referral letter were considered for processing. Missing data from the questionnaire were not replaced by imputation of any kind, whereas all data gathered from the EHRs were included in the analysis. Subsequently, this extended data set (ED) was randomly separated into an evaluation data set (with 50 samples per treatment category) and a training data set consisting of the remaining data set entries (see Table 1 for descriptive statistics per treatment category). Table 1 indicates that the given data set is unbalanced, a common challenge for training ML-based CDSSs. Using original samples for the evaluation data set ensured that our findings were meaningful, and the equal distribution of samples among treatment categories in this evaluation data set ensured fair classification results without considering the original distribution among treatment categories. With the evaluation data set being balanced, sample distribution among the considered treatment categories in the training set (for the original sample distribution among classes, see Table 1) was balanced via the synthetic minority oversampling technique (SMOTE) [14] (with k-nearest neighbors=5 and strategy=auto). SMOTE is a data augmentation approach that augments data entries for minority categories [15], thereby supporting multiclass resampling using a one-versus-rest scheme [14]. The balancing via SMOTE ensures that each treatment category is considered equally relevant.

All previous processing steps were performed in the ED. A basic data set (BD) was generated by duplicating the ED and removing additional EHR parameters to investigate the ED’s relevance. Both data sets were z-score-normalized (separately for training, validation, and evaluation) via the *scikit-learn* library of Python.

#### Natural Language Processing

To quantify and embed the EHR data into the BD, natural language processing (NLP) was applied. NLP is a technique used to extract meaning from qualitative data, which can be used to structure large amounts of unstructured, qualitative data. It is well accepted that in referral letters, meaningful information is included, but automatic extraction can only be achieved via NLP. In our programmed processing pipeline, we conducted named entity recognition (NER) via specific words (see Tables 2 and 3) via the regular expression operations module *RE-Package* of Python. With RE-Package, regular words or expressions were identified, which are a sequence of characters that specify a search pattern for defined code words in between which additional characters might occur.

The American Standard Code for Information Interchange (ASCII)–encoded referral letter texts were analyzed by trained clinical personnel regarding the description of the following data: referral reason and patient goals

**Table 2:** Referral reason features, including search term examples, meaning, and statistics of occurrence (overall referral letters) in all detected referral reason findings. For a comprehensive list of code words, see Multimedia Appendix 1.

Feature	Meaning	Dutch code word example (English translation)	Occurrences, n (%)
SecondOpinionReRe^a^	Referral for a second opinion/diagnostics	*aanvullend onderzoek* (additional investigation)	383 (29)
AnesthesiologyReRe	Referral for anesthesiology	*blockade* (nerve block)	180 (14)
RehabReRe	Referral for interdisciplinary rehabilitation	*revalidatie* (rehabilitation)	442 (34)
AdviceReRe	Could you give advice to the patient?	*advies* (advice)	141 (11)
OptionsReRe	Are there treatment options?	*behandelbare opties* (options for treatment)	172 (13)

^a^ReRe: referral reason.

**Table 3:** Patient goal features, including search term examples, meaning, and statistics of occurrence among all recognized patient questions for help finding. For a comprehensive list of code words, see Multimedia Appendix 2.

Feature	Meaning	Dutch code word example (English translation)	Occurrences, n (%)
MoreCausePaG^a^	More diagnostics	*2e mening* (2nd opinion)	128 (22)
PainRedPaG	Pain reduction	*pijn* (pain)	241 (42)
BetterFuncPaG	Better functioning	*behandelopties* (treatment options)	188 (32)
AdvicePaG	Advice	*advies* (advice)	23 (4)

^a^PaG: patient goal.

RE-Package was used to find free-text words related to the referral reasons and patient goals, which were stated in Dutch. The corresponding code words are summarized in Multimedia Appendices 1 and 2. Corresponding information was extracted via an NLP processing pipeline. In a preprocessing step of the processing pipeline, ambiguous wordings were unified. To create this pipeline, referral letters were manually studied by 2 authors (RS and PS) independently to determine relevant types of referral reasons and patient goals and corresponding code words indicating them. All words were classified. The resulting categories of referral reasons and patient goals are summarized in Tables 2 and 3, respectively, with their number of occurrences.

The initial textual data were transferred to JavaScript Object Notation (JSON) data interchange format via the *Panda* library [16]. Filtering of referral letters was implemented via RE-Package [17], resulting in lists of referral letters that contained the corresponding code words. Redundant framings were unified via regular expressions. Subsequently, relevant text segments, especially the ones covering the referral reasons and patient goals, were identified via regular expressions in the referral letters’ extracted text. Within these text segments, the corresponding code words from the EHRs (see Tables 2 and 3) were screened via regular expressions. All referral reason features were extracted from the referral reason text; if multiple referral reason entries were available per patient, all were considered. Similarly, all patient goal features were extracted from the patient questions for help text; if multiple patient goal entries per patient were identified, all were considered (eg, “I want a solution for my pain, and I want to know what is wrong with my back.”). Correspondingly, 4 of the 5 new referral reason features and 4 new patient goal features were included in the data set. Henceforth, we refer to all parameters extracted from the referral letters (specifically the referral reasons and patient goals) as “referral letter parameters.”

To ensure the interpretability of the ML models and of the effect of the additional referral parameters, we excluded any feature engineering but kept the input parameters as they were. By not constructing features by recombining the input parameters, we investigated the relevance and benefit of each feature and ensured high explainability of the network.

#### Feature Selection

To identify the relevance of the ED compared to the BD and to limit the number of features so that they could be gathered in a questionnaire in standard clinical use, we applied the ReliefF feature selection algorithm [18] to both the ED and the BD. For feature selection, only the entries in the training sets were considered before applying SMOTE.

The general aim of feature selection was to identify a subset of features by which the data space spanned as much as possible (including as much variety as possible), while data points in the same class (an alternative naming of the treatment category) remained as small as possible. The ReliefF algorithm [18] is a supervised feature selection method for multiclass problems that is robust against incomplete and noisy data, considering the k-nearest neighbors (kNNs) [19]. It is a multivariate filter that completely ranks individual features according to their relevance for class separation in the context of other features throughout the observations in the training set [20]. ReliefF calculates a feature score based on the differences among feature (parameter) values between neighboring instances. By considering the differences in features among neighboring samples of class hits (NH) that belong to the same class and neighboring samples of class misses (NM), the algorithm can calculate the relevance each feature contributes to determining class membership. With the original Relief function supporting only biclass problems, ReliefF supports multiclass search and considers k-near misses per class.

The feature relevance score (S) for a particular feature was consequently calculated as follows, with x representing the feature value, abs() denoting the absolute value, and n representing the total number of instances in the data set:

S = sum(abs(x – NH) – abs(x – NM))/n

Correspondingly, each feature’s weight update (W) was calculated as follows, with M representing the total number of features:

W = W – (abs(x – NH) – abs(x – NM))/(n × M)

These equations were used iteratively, and the feature scores were averaged over all instances.

By applying ReliefF, the input parameters were ranked in accordance with their relevance for class separation. The ReliefF algorithm was configured for m=n (to all numbers of training samples) [21], the number of kNNs=50 [19], and the feature relevance threshold τ=0.7 [22] for selecting features as common parameter settings.

#### Training and Machine Learning

To analyze the benefit of the referral letter parameters regarding a treatment path decision, ML models were trained for both data sets, the BD and the ED, after feature selection. For achieving good results in small, unbalanced data sets, such as the given ones, and allowing for interpretability of the results, the kNN algorithm, support vector machine (SVM), and multilayer perceptron (MLP) models were used in the evaluation. Being one of the first studies to apply both quantitative and qualitative (NLP-extracted) data, we decided to focus on applying well-established neural network architectures, as they are well understood and especially allow interpretability of the classification processes, which is essential when investigating new data types. We contacted the clinicians, and they confirmed the high criticality of having interpretability of the neural networks, which should lead to applicable or treatable options for the patient.

Another advantage is that these classical ML approaches are much better suited than deep neural networks for small, unbalanced data sets. In recent works, we found that in smaller data sets, the classical approaches perform similarly well as deep learning networks.

**Figure 1:**
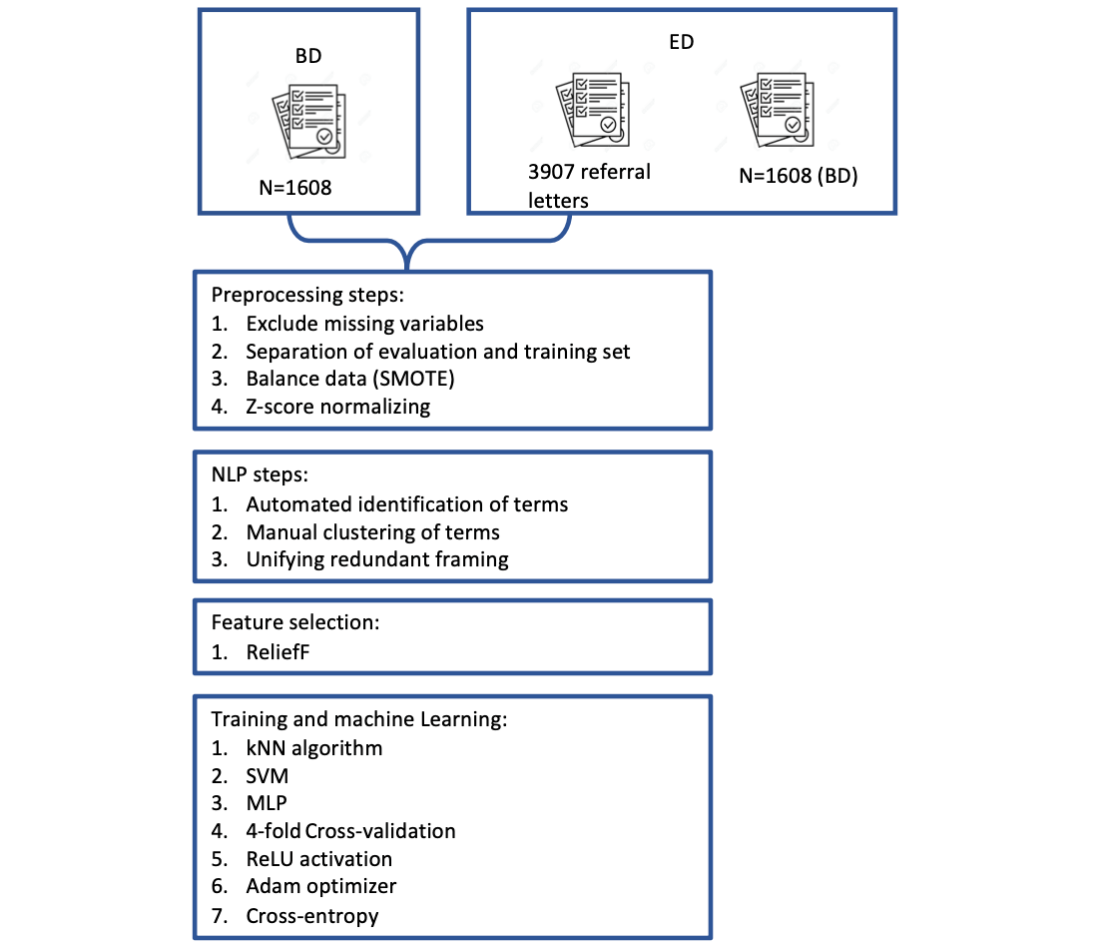
Process to reach the final ML algorithm. BD: basic data set; ED: extended data set; kNN: k-nearest neighbor; ML: machine learning; MLP: multilayer perceptron; ReLU: rectified linear unit; SMOTE: synthetic minority oversampling technique; SVM: support vector machine.

For training of the models, 4-fold cross-validation, the rectified linear unit (ReLU) activation function, the Adam optimizer, and the cross-entropy loss function via scikit-learn were used. Further implementation details can be found in the code available in Ref. [23]. For training and evaluation, an AMD Ryzen 9 5900X with 64GB RAM and NVIDIA GeForce RTX 3070 was used running 64-bit Windows 11 Pro, Python 3.8, Keras 2.4.3, Keras-Preprocessing 1.1.2, Keras-tuner 1.0.1, pandas 1.1.1, scikit-learn 0.23.2, skrebate 0.62, and TensorFlow 2.4.1. A complete overview of all the steps is presented in Figure 1.

### Statistical Analysis

The data set was characterized by sociodemographic and health-related data and distribution characteristics of the referral reasons and patient goals on the treatment categories. The relevance of the referral letter parameters was investigated based on the ReliefF ranking for both data sets (ED and BD). The accuracy of the ML models was evaluated via the *F*_1_-score and confusion matrices.

The *F*_1_-score is a commonly used metric to evaluate the performance of a classification model via precision (P) and recall (R). Precision measures the ratio of correctly predicted true-positive (TP) instances to the total predicted TP instances and false-positive (FP) instances:

P = TP/(TP + FP)

Recall measures the ratio of correctly predicted TP instances to the total actual TP instances, thus including the number of incorrectly predicted false-negative (FN) instances:

R = TP/(TP + FN)

In the following equation, the *F*_1_-score represents the harmonic mean of precision and recall and thus represents a balanced evaluation of the model’s performance:

*F*_1_-score = 2 × (P × R)/(P + R)

The *F*_1_-scores were averaged over 4 separate training runs.

Confusion matrices were plotted for the BD and ED classifiers. A confusion matrix summarizes prediction results on a classification problem in a visual manner. By analyzing confusion matrices, we can investigate where the classifier misperforms regularly, indicating lower feature variability.

## Results

### Study Cohort

We originally included 1608 patients and corresponding 3907 letters in the data set. The age of the patients ranged from 18 to 66 years, with a mean age of 46 (SD 12.93) years, and 914 (57.2%) patients were female and on average 45 (SD 13.3) years younger than men, who had a mean age of 48 (SD 12.4) years. Of the 1608 patients, 1341 (83.4%) had referral letters included. Of these, 1209 (90.2%) patients were included because their referral letters included referral reasons (n=1062, 87.8%, cases) or patient goals (n=746, 61.7%); 160 referral letters contained only patient goals. Multiple referral letters were available and included for 1012 (83.7%) patients, with redundant referral letters for 1006 (75%) patients with referral letters. Once balanced, the training set included 521 entries per treatment category.

The training data set included 1009 patients, who were distributed among the treatment categories, as shown in Table 1. Within the training and evaluation sets, Tables 4 and 5 present the category-specific extracted referral reasons and corresponding patient goals, respectively.

**Table 4:** Category-specific statistics of the available referral reasons specified within the referral letters, considered in the training and evaluation data sets. Sample distribution among categories and the corresponding sums and overall percentage distribution are shown. “Referral reason” categories refer to Table 2.

Referral reason category	Treatment: rehabilitation (1), n (%)			Treatment: anesthesiology (2), n (%)			Treatment: neurosurgery (3), n (%)			Treatment: no treatment (0), n (%)	
	Training	Evaluation	Training		Evaluation	Training		Evaluation	Training		Evaluation
SecondOpinionReRe^a^ (1)	167 (13)	17 (1)	56 (4)		24 (2)	33 (3)		21 (2)	76 (6)		15 (1)
AnesthesiologyReRe (2)	22 (2)	3 (0)	80 (6)		34 (3)	6 (0)		9 (1)	6 (0)		3 (0)
RehabReRe (3)	302 (23)	28 (2)	30 (2)		10 (1)	8 (1)		5 (0)	47 (4)		7 (1)
AdviceReRe (4)	71 (5)	7 (1)	20 (2)		5 (0)	7 (1)		4 (0)	24 (2)		3 (0)
OptionsReRe (5)	85 (6)	9 (1)	36 (3)		5 (0)	10 (1)		6 (0)	16 (1)		5 (0)
Sum	647 (48.9)	64 (4.8)	222 (16.8)		78 (5.9)	64 (4.8)		45 (3.4)	169 (12.8)		33 (2.5)

^a^ReRe: referral reason.

**Table 5:** Category-specific statistics of the available patient goal specified within the referral letters, considered in the training and evaluation data sets. Sample distribution among categories and the corresponding sums and overall percentual distribution are shown. “Patient goal” categories refer to Table 3.

Patient goal category	Treatment: rehabilitation (1), n (%)			Treatment: anesthesiology (2), n (%)			Treatment: neurosurgery (3), n (%)			Treatment: no treatment (0), n (%)	
	Training	Evaluation	Training		Evaluation	Training		Evaluation	Training		Evaluation
MoreCausePAG^a^ (1)	78 (14)	5 (1)	9 (2)		1 (0)	2 (0)		2 (0)	30 (5)		1 (0)
PainRedPAG (2)	126 (22)	9 (2)	16 (3)		5 (1)	4 (1)		2 (0)	65 (11)		14 (2)
BetterFuncPAG (3)	107 (19)	8 (1)	13 (2)		5 (1)	4 (1)		1 (0)	44 (8)		6 (1)
AdvicePAG (4)	13 (2)	1 (0)	1 (0)		0	0		0	5 (1)		3 (1)
Sum	324 (55.9)	23 (4.0)	39 (6.7)		11 (1.9)	10 (1.7)		5 (0.9)	144 (24.8)		24 (4.1)

^a^PaG: patient goal.

### Feature Selection and Information Gain

The number of features to be considered in both cases was determined to be 29 for the BD and 30 for the ED based on the applied feature relevance threshold of 0.7. Table 6 summarizes the selected features in the order of decreasing information gain, indicating the information gain of the AnesthesiologyReRe and RehabReRe referral reason features as the second- and fourth-most important referral features. Including the referral letter parameters in the BD did not change the general feature-ranking types between the data sets.

**Table 6:** Ranked features based on ReliefF feature selection for the BD^a^ and the ED^b^.

Rank	ED			BD	
	Feature	ReliefF	Feature		ReliefF
1	Have you been absent from your work in the past 4 weeks because you were sick?	0.127	Have you been absent from your work in the past 4 weeks because you were sick?		0.128
2	AnesthesiologyReRe^c,d^	0.118	Have there been days in the past 4 weeks when you worked but suffered from physical or psychological problems during your work?		0.109
3	Have there been days in the past 4 weeks when you worked but suffered from physical or psychological problems during your work?	0.107	How many hours a week do you work? Add together all the hours for which you are paid.		0.103
4	RehabilitationReRe^d^	0.106	Have you used injections (eg, epidural steroid or facet injections)?		0.103
5	How many hours a week do you work? Add together all the hours for which you are paid.	0.104	Have you ever had a low back operation?		0.101
6	Have you used injections (eg, epidural steroid or facet injections)?	0.103	Have you used exercise therapy?		0.093
7	Have you ever had a low back operation?	0.102	Do you think, based on your current health situation, you will still be able to work for the next 2 years?		0.092
8	Leg pain is dominant.	0.093	Regarding your work in general, how pleased are you with your job as a whole, considering everything?		0.091
9	Do you think, based on your current health situation, you will still be able to work for the next 2 years?	0.092	Leg pain is dominant.		0.090
10	Have you used exercise therapy?	0.092	Is your work recognized and appreciated by management?		0.085
11	Regarding your work in general, how pleased are you with your job as a whole, considering everything?	0.088	How often is your immediate superior willing to listen to your work-related problems?		0.085
12	Is your work recognized and appreciated by management?	0.085	Are you treated fairly at your workplace?		0.085
13	How often is your immediate superior willing to listen to your work-related problems?	0.084	How often are your colleagues willing to listen to your work-related problems?		0.084
14	Are you treated fairly at your workplace?	0.084	Is your work meaningful?		0.084
15	How often are your colleagues willing to listen to your work-related problems?	0.084	Are conflicts resolved in a fair way?		0.083
16	Are conflicts resolved in a fair way?	0.083	How often do you get help and support from your nearest superior?		0.082
17	Is your work meaningful?	0.082	Do you have enough time for your work tasks?		0.080
18	How often do you get help and support from your nearest superior?	0.082	Have you used opioid painkillers (prescription medications, such as Vicodin, Lortab, Norco, hydrocodone, codeine, Tylenol, Fentanyl, Duragesic, MS Contin, Percocet, Tylox, OxyContin, oxycodone, methadone, tramadol, Ultram, or Dilaudid)?		0.080
19	Do you have enough time for your work tasks?	0.079	Do you have to relate to other people’s personal problems as part of your work?		0.079
20	How often do you get help and support from your colleagues?	0.079	How often do you get help and support from your colleagues?		0.079
21	Is your workload unevenly distributed so it piles up?	0.078	Do you feel that the work you do is important?		0.079
22	Do you feel that the work you do is important?	0.078	Is your workload unevenly distributed so it piles up?		0.078
23	Do you have to relate to other people’s personal problems as part of your work?	0.078	Do you feel that your work drains so much of your energy that it has a negative effect on your private life?		0.076
24	Have you used opioid painkillers (prescription medications, such as Vicodin, Lortab, Norco, hydrocodone, codeine, Tylenon, Fentanyl, Duragesic, MS Contin, Percocet, Tylox, OxyContin, oxycodone, methadone, tramadol, Ultram, or Dilaudid)?	0.076	Can you influence the amount of work assigned to you?		0.076
25	Do you feel that your work drains so much of your energy that it has a negative effect on your private life?	0.076	Does your work put you in emotionally disturbing situations?		0.075
26	Do you have to work fast?	0.075	Do you have to work fast?		0.075
27	Would you ask a good friend to apply for a position at your workplace?	0.074	Would you ask a good friend to apply for a position at your workplace?		0.075
28	Does your work put you in emotionally disturbing situations?	0.074	Do you get behind with your work?		0.071
29	Can you influence the amount of work assigned to you?	0.074	Do you feel that your work takes so much of your time that it has a negative effect on your personal life?		0.070

^a^BD: basic data set.

^b^ED: extended data set.

^c^ReRe: referral reason.

^d^AnesthesiologyReRe and RehabReRe were the second- and fourth-most important referral reason features.

### Accuracy of Machine Learning Models

In general, *F*_1_-scores were low for both the BD and the ED, with values ranging from 0.28 to 0.54. Comparing the *F*_1_-scores between the BD and the ED showed an increase of up to 19.5% for the *F*_1_-score when considering the additional referral reason features (see Table 7).

**Table 7:** Table 7.F1-scores of the ML^a^ methods for both data sets.

ML method	*F*_1_-scores for the ED^b^ (top 30)	*F*_1_-scores for the BD^c^ (top 29)
SVM^d^	0.535	0.355
kNN^e^	0.505	0.315
MLP^f^	0.470	0.275

^a^ML: machine learning.

^b^ED: extended data set.

^c^BD: basic data set.

^d^SVM: support vector machine.

^e^kNN: k-nearest neighbor.

^f^MLP: multilayer perceptron.

**Figure 2:**
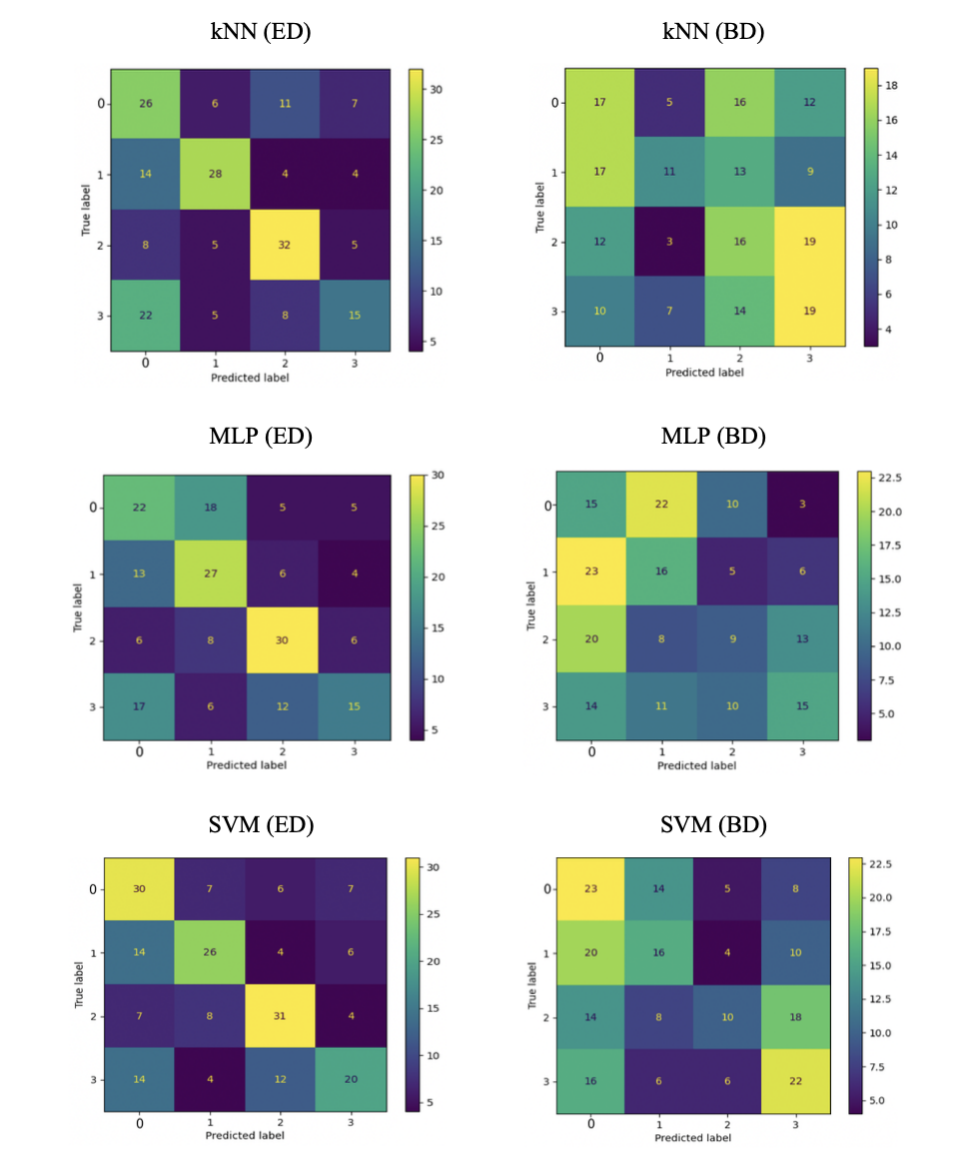
Confusion matrices for data set–specific ML models for the ED with 30 features and the BD with 29 features. BD: basic data set; ED: extended data set; kNN: k-nearest neighbor; ML: machine learning; MLP: multilayer perceptron; SVM: support vector machine.

In addition, the confusion matrices of the SVM, kNN, and MLP among both conditions are shown in Figure 2. Each column and row represent 1 treatment category. The rows represent the actual (annotated) categories. The columns represent the predicted treatment categories by classifier. The numbers in the cells of a confusion matrix represent the number of samples that have been predicted for the column but are annotated for the row. High numbers in the cells on the diagonal line from the upper left to the lower right cell represent TPs (correctly predicted samples). The numbers in the surrounding cells represent faulty detections. Consequently, yellow and green colors in the diagonal are good, and blue in the surrounding is good as it represents a low number of misses. Yellow and green in the surroundings indicate a stronger error.

The confusion matrices highlighted the benefit of the additional referral letter parameters, as the ED had a much higher agreement among the predicted and true labels than the BD, which is indicated by having more yellow and green on the diagonal upper left to lower right and more blue surrounding cells in the ED compared to the BD.

## Discussion

### Principal Findings

The results of this study indicate that quantitative self-report data from patients with LBP can be enriched by unstructured and qualitative data collected from referral letters as part of EHRs using NLP. NLP appears as a feasible option, and we found that the performance of our ML models increased significantly by up to 19.5%. When considering the relevance of referral reasons from EHRs, especially the referral reasons for the AnesthesiologieReRe and RehabReRe categories were relevant, being the second- and fourth-most important referral features of the 30 relevant items. Considering the occurrence of corresponding fields, in 180 referrals, there was a question for the anesthesiology treatment, whereas 228 patients were actually triaged to anesthesiology. For the 571 patients triaged to rehabilitation, in 442 occurrences, the referral reason indicated rehabilitation as well, with an agreement of 38.4%, indicating that the referrer influences the triaging process for the patient in secondary and tertiary care.

What we could not find were direct referrals to a neurosurgery intervention, probably because this is unusual in the Netherlands. More common is to refer patients with a referral reason to more diagnostics by, for example, a neurologist who will, in positive cases, refer them to neurosurgery. With neurosurgery being a small sample in our data set and the code words resulting from visual inspection of the data set, that might relate to one of the following:

- The suggestion is less specific within the text and may also be stated as “Please, more diagnosis” or “Are there treatment options?”
- The small data sample is less representative of the neurosurgery category because only limited cases included neurosurgery and we used the unbalanced data set in the feature selection.

Although the patient goals for help in the domains of “pain reduction” with 42% and “better functioning” with 32% cover the majority of clusters, none of these goals could be directly associated with the triaging decision.

Evaluating the effect of the 2 included referral reasons on the accuracy of the ML models, classic ML approaches (ie, SVM, kNN, and MLP) were chosen as these are reportedly more suitable for such small data sets than deeper neural network architectures. The corresponding results in Table 6 clarify the relevance of the referral reasons in achieving a significantly improved triaging accuracy ranging between 18% and 19.5%. To investigate the effect, these 2 referral reasons hold for ML-supported decision support, and we investigated the confusion matrices (Figure 2). In all cases, we saw a significantly enhanced accuracy of the predicted and annotated treatment categories when considering the referral reasons (ED) compared to not considering them (BD).

It can be concluded that for clinical significance, EHR data can hold valuable information for the prediction of triaging patients with LBP to their treatment. Especially the wording and referrals of the general physician or secondary care specialist significantly and relevantly increase ML model fits. The goals and desires of the patient do not contribute to the prediction of triaging.

### Limitations

There are a number of limitations that should be addressed. First, the unbalanced data set and the overrepresented rehabilitation intervention category held 571 (47%) cases, while neurosurgery held only 109 (9%) cases. Considering the referral reasons, we found a similar unbalanced distribution. These unbalanced data might have led to an overrepresented data accuracy of rehabilitation compared to neurosurgery. Therefore, we applied SMOTE data augmentation for the ML model training. With only 59 data entries being available for training the treatment category neurosurgery, nearly 90% of the corresponding training entries were augmented. In addition, only a limited number of referral letters and the included referral reasons and patient goals were available for this treatment category. Consequently, the meaningfulness of the findings regarding the treatment category neurosurgery should be confirmed in another data set. In contrast to this minority treatment category, the results of the other treatment categories can be assumed to be representative.

A second limitation is the number of missing data and letters that we could derive from the EHRs. Although most could be retrieved, it appeared impossible to retrieve all letters, and in some cases, letters may have bene be missing or may not have been uploaded properly. In addition, although cases were missing, it may have led to higher external validity. In many real-world clinical situations, data sets are incomplete. Therefore, we decided to include what we could derive following a commonly applied approach.

Lastly, we could, based on these baseline data, not conclude whether the triaging to the treatment was the correct triaging in terms of the treatment with the highest benefit to the patient and whether the patient indeed was successfully treated. Future longitudinal studies may lead to a better understanding of the use of ML-based clinical decision-making in patients with LBP, for example, by including the results of treatments.

### Strengths

In addition to the quantitative research questions that we answered, this study contributes to the discussion of the meaningfulness of the general physician’s referral reasons and patient goals in a group of patients with LBP. Correspondingly, the resulting input feature ranking of both data sets is expected to represent the information relevance of the feature and might indicate the impact, referral reasons, and patient goals have on treatment category (class) selection.

Considering the generalizability toward an additional language or to other LBP-related questionnaires should be straightforward. Although adjustments of search terms and potential alternative document structures are required, the proposed approach allows quick adaptability. Covering additional diseases or using additional or different questionnaires will require clinical and methodological knowledge of the corresponding domain experts.

### Conclusion

Among the ReliefF-prioritized features, 2 referral reason features were highly relevant, and their consideration increased the *F*_1_-score accuracy of the models by up to 19.5%. The results were confirmed by visual inspection of confusion matrices, although the overall performance of the ML models remains low and they cannot be clinically applied at this moment.

## Appendix

Multimedia Appendix 1 Referral reason categories and code words.

Multimedia Appendix 2 Patient goal categories and code words.

## Abbreviations

BD: basic data set
CDSS: clinical decision support system
DSS: decision support system
ED: extended data set
EHR: electronic health record
FN: false negative
FP: false positive
kNN: k-nearest neighbor
LBP: low back pain
ML: machine learning
MLP: multilayer perceptron
NH: neighboring samples of class hits
NLP: natural language processing
NM: neighboring samples of class misses
PA: physician assistant
ReLU: rectified linear unit
SMOTE: synthetic minority oversampling technique
SVM: support vector machine
TP: true positive

## References

[1] Maher C, Underwood M, Buchbinder R (2017). Non-specific low back pain. Lancet.

[2] Foster NE, Anema JR, Cherkin D, Chou R, Cohen SP, Gross DP, Ferreira PH, Fritz JM, Koes BW, Peul W, Turner JA, Maher CG, Buchbinder R, Hartvigsen J, Cherkin D, Foster NE, Maher CG, Underwood M, van Tulder M, Anema JR, Chou R, Cohen SP, Menezes Costa L, Croft P, Ferreira M, Ferreira PH, Fritz JM, Genevay S, Gross DP, Hancock MJ, Hoy D, Karppinen J, Koes BW, Kongsted A, Louw Q, Öberg B, Peul WC, Pransky G, Schoene M, Sieper J, Smeets RJ, Turner JA, Woolf A (2018). Prevention and treatment of low back pain: evidence, challenges, and promising directions. Lancet.

[3] Foster NE, Hill JC, O'Sullivan P, Hancock M (2013). Stratified models of care. Best Pract Res Clin Rheumatol.

[4] Linton SJ, Halldén K (1998). Can we screen for problematic back pain? A screening questionnaire for predicting outcome in acute and subacute back pain. Clin J Pain.

[5] Hill JC, Dunn KM, Lewis M, Mullis R, Main CJ, Foster NE, Hay EM (2008). A primary care back pain screening tool: identifying patient subgroups for initial treatment. Arthritis Rheum.

[6] da Silva T, Macaskill P, Kongsted A, Mills K, Maher CG, Hancock MJ (2019). Predicting pain recovery in patients with acute low back pain: updating and validation of a clinical prediction model. Eur J Pain.

[7] d’Hollosy W, van Velsen L, Poel M, Groothuis-Oudshoorn C, Soer R, Stegeman P, Hermens H (2020). Applying machine learning on patient-reported data to model the selection of appropriate treatments for low back pain: a pilot study.

[8] van Hooff ML, van Loon J, van Limbeek J, de Kleuver M (2014). The Nijmegen decision tool for chronic low back pain. Development of a clinical decision tool for secondary or tertiary spine care specialists. PLoS One.

[9] Dutmer AL, Schiphorst Preuper HR, Soer R, Brouwer S, Bültmann Ute, Dijkstra PU, Coppes MH, Stegeman P, Buskens E, van Asselt ADI, Wolff AP, Reneman MF (2019). Personal and societal impact of low back pain: The Groningen Spine Cohort. Spine (Phila Pa 1976).

[10] EuroQol Group (1990). EuroQol--a new facility for the measurement of health-related quality of life. Health Policy.

[11] Deyo RA, Dworkin SF, Amtmann D, Andersson G, Borenstein D, Carragee E, Carrino J, Chou R, Cook K, DeLitto A, Goertz C, Khalsa P, Loeser J, Mackey S, Panagis J, Rainville J, Tosteson T, Turk D, Von Korff M, Weiner DK (2014). Report of the NIH Task Force on research standards for chronic low back pain. Spine.

[12] El Fassi M, Bocquet V, Majery N, Lair ML, Couffignal S, Mairiaux P (2013). Work ability assessment in a worker population: comparison and determinants of Work Ability Index and Work Ability score. BMC Public Health.

[13] Pejtersen JH, Kristensen TS, Borg V, Bjorner JB (2010). The second version of the Copenhagen Psychosocial Questionnaire. Scand J Public Health.

[14] Chawla NV, Bowyer KW, Hall LO, Kegelmeyer WP (2002). SMOTE: synthetic minority over-sampling technique. J Artif Intell Res.

[15] Over-sampling methods. Imbalanced learn.

[16] Bray T (2017). STD 90, RFC 8259: the JavaScript Object Notation (JSON) Data Interchange Format. RFC Editor.

[17] re — Regular expression operations. Python Software Foundation.

[18] Robnik-Šikonja M, Kononenko I (1997). An adaptation of Relief for attribute estimation in regression.

[19] Robnik-Šikonja M, Kononenko I (2003). Theoretical and empirical analysis of ReliefF and RReliefF. Mach Learn.

[20] Guyon I, Nikravesh M, Gunn S, Zadeh LA (2006). Feature Extraction.

[21] Kononenko I (1994). Estimating attributes: analysis and extensions of RELIEF.

[22] Kira K, Rendell LA (1992). The feature selection problem: traditional methods and a new algorithm.

[23] NLP_Triaging Source Code. GitHub.

