# Supplementary figures and images for: Natural Language Processing of Referral Letters for Machine Learning–Based Triaging of Patients With Low Back Pain to the Most Appropriate Intervention: Retrospective Study
Source: J Med Internet Res. 2024 Jan 30;26:e46857. doi: 10.2196/46857 (PMC10865208; doi:10.2196/46857)

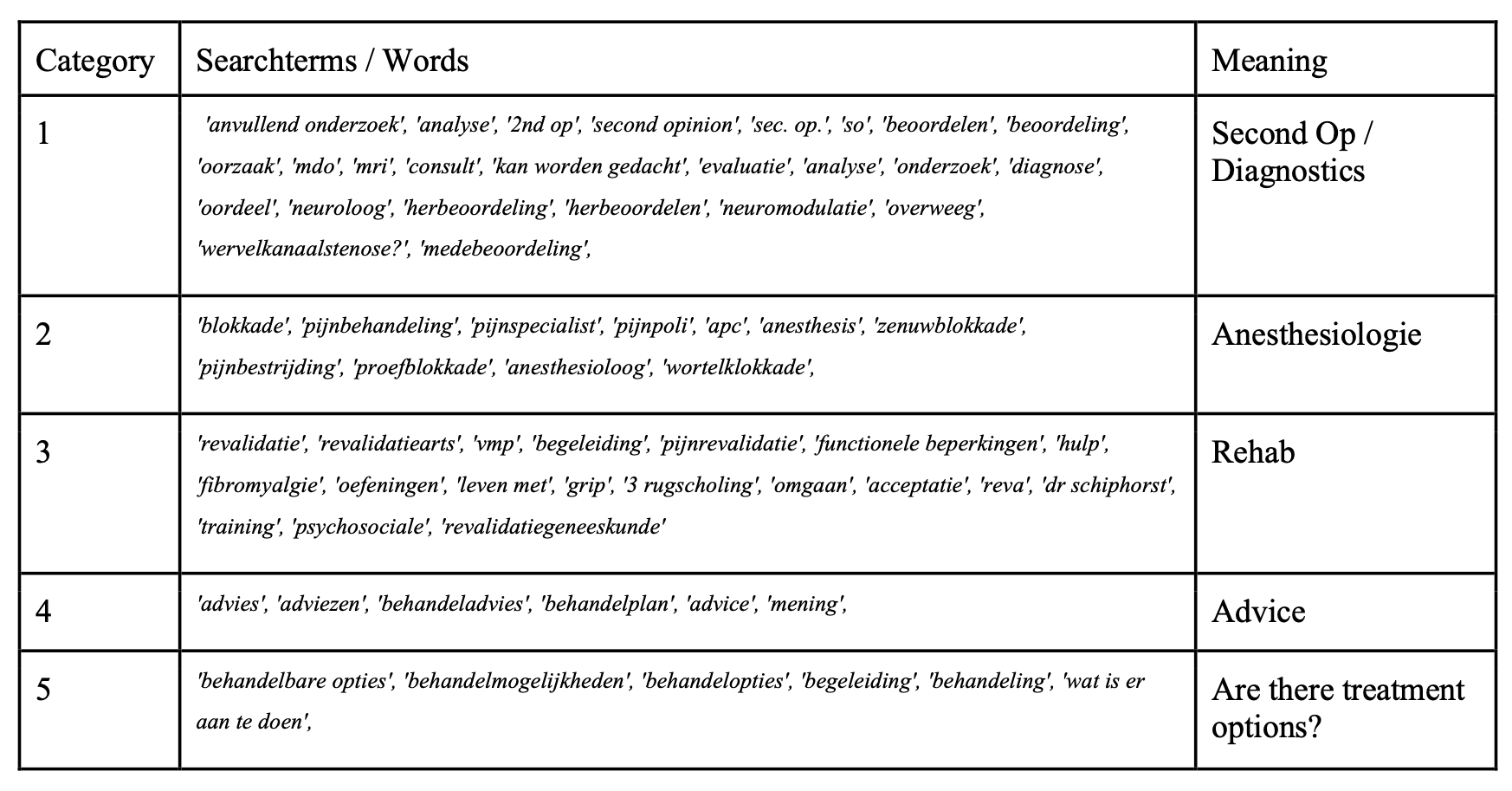

Supplement: Multimedia Appendix 1 [file jmir_v26i1e46857_app1.png]

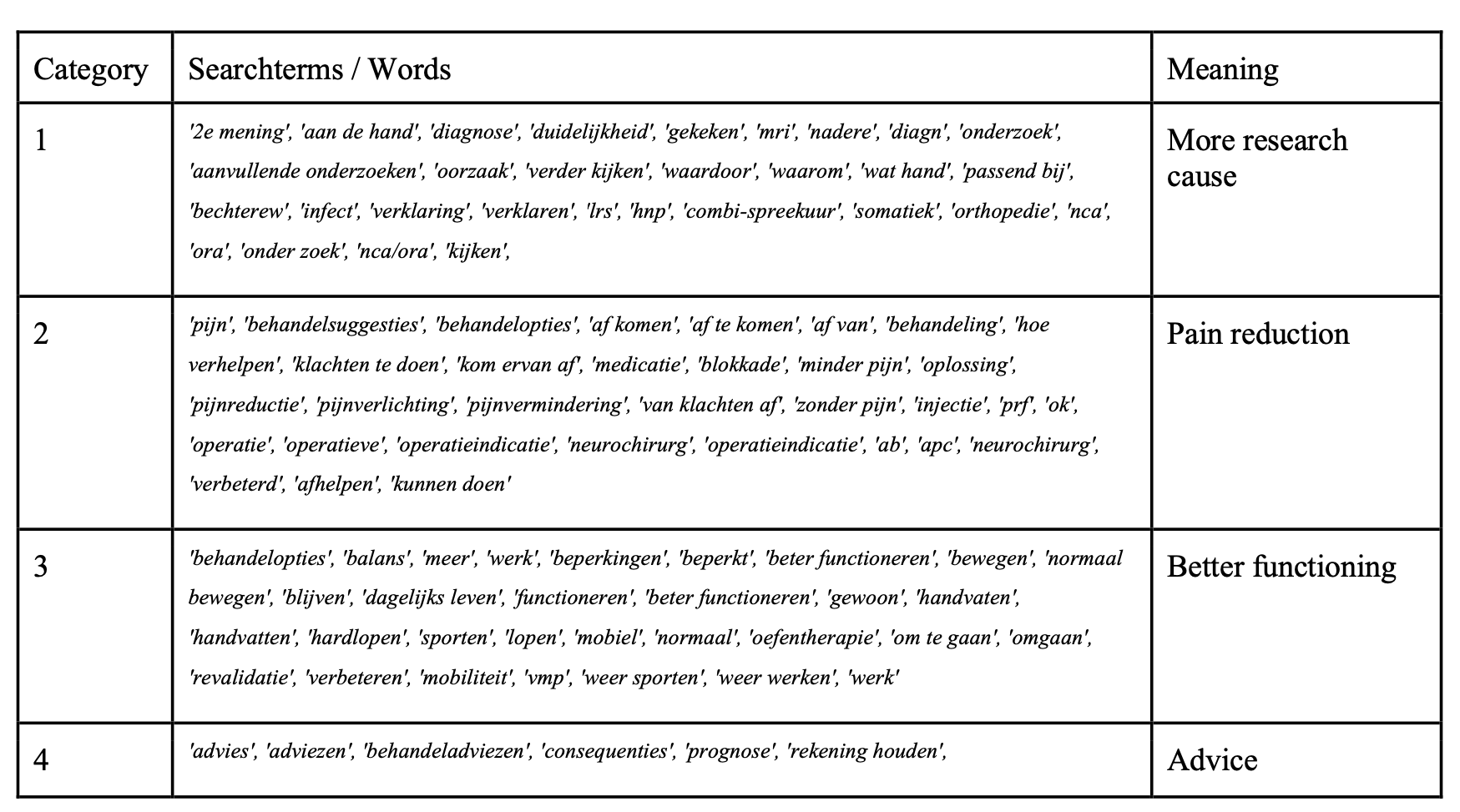

Supplement: Multimedia Appendix 2 [file jmir_v26i1e46857_app2.png]
